# Evaluating CBCT-Guided Adaptive Radiotherapy for Pancreatic Cancer Using Synthetic CBCT Data

**DOI:** 10.3390/curroncol32020060

**Published:** 2025-01-23

**Authors:** Sven Olberg, Leah L. Thompson, Hannah J. Roberts, Jennifer Y. Wo, Theodore S. Hong, John Wolfgang, Clemens Grassberger, Jennifer Pursley

**Affiliations:** 1 Department of Radiation Oncology, Massachusetts General Hospital, Boston, MA 02114, USA; 2 Department of Radiation Oncology, University of Washington, Seattle, WA 98195, USA

**Keywords:** adaptive radiotherapy, CBCT guidance, pancreatic cancer

## Abstract

Ethos adaptive radiotherapy is employed frequently in the pelvis to improve treatment accuracy by adapting to daily anatomical changes. The use of this CBCT-guided platform for abdominal treatments is made challenging by motion-related image artifacts that are detrimental to the Ethos auto-contouring process. We present a preliminary in silico study enabled by synthetic CBCT data of Ethos adaptive radiotherapy for pancreatic cancer. Simulation CT and daily CBCT images were collected from nonadaptive patients treated on Ethos. Contoured CBCTs drove structure-guided deformable registration from the CT to daily CBCTs, providing an approximate daily CT used to produce synthetic CBCT data. Two adaptive workflows were simulated using an Ethos emulator. Over 70 fractions across 10 patients in a solely deformation-based workflow, PTV prescription coverage increased by 23.3±9.4% through plan adaptation. Point doses to the stomach were reduced by 10.2±9.3%. Ultimately, un-adapted plans satisfied target coverage and OAR constraints in 0% and 6% of fractions while adapted plans did so in 80% of fractions. Anatomical variation led to poor performance in rigidly aligned un-adapted plans, illustrating the promise of Ethos adaptive radiotherapy in this region. This promise is balanced by the need for artifact reduction and questions regarding auto-contouring performance in the abdomen.

## 1. Introduction

Adaptive radiation therapy (ART) workflows have been widely employed in sites like the general pelvis with the aim of improving treatment outcomes by responding to changes in patient anatomy throughout the course of treatment [1,2,3,4,5,6,7,8,9,10,11]. There is considerable interest in bringing adaptive workflows into the abdomen, where targets are often closely surrounded by radiosensitive and highly mobile gastrointestinal organs at risk (OARs) [1,2,12,13,14]. For pancreatic cancer patients specifically, these surrounding critical structures include the stomach, duodenum, and bowel, which all demonstrate significant changes in volume and position relative to the target that are observable on a daily basis [15,16]. An online ART workflow applied in this setting would allow for the modification of a treatment plan to accommodate these daily anatomical variations, improving target coverage or OAR sparing, for example, or facilitating dose escalation in an iso-toxic approach [5,17].

The adoption of these online ART workflows for various disease sites has been driven in large part by the commercial availability of platforms designed with a focus on ART, including the magnetic resonance imaging (MRI)-guided MRIdian [18,19] (ViewRay, Denver, CO, USA) and Unity [20,21] (Elekta Solutions AB, Stockholm, SE, USA) systems along with the cone beam computed tomography (CBCT)-guided Ethos [22] (Varian Medical Systems, Palo Alto, CA, USA). These MRI-guided platforms offer the distinct advantage of improved soft tissue contrast compared to CT imaging, which is particularly valuable for structure delineation in the abdomen, as well as real-time imaging capabilities that allow for beam gating during treatment delivery [23,24]. However, image acquisition times ranging from 30 s to several minutes and MR-safety requirements can be limiting in this setting [25,26]. As such, the decision between the MRI- and CBCT-guided platforms is not a straightforward one; indeed, the use of CBCT, which has long been the gold standard for fast, volumetric image guidance in radiation therapy, makes the Ethos system an attractive alternative for establishing an ART program [27].

In contrast to these existing MR-based workflows in which OAR contours are propagated via image registration, the Ethos workflow relies primarily on an auto-contouring step in which ‘influencer’ structures—site-specific organs that influence the shape of common targets—are contoured via a deep neural network [28,29,30]. This capability enables a streamlined adaptive workflow in relatively static sites like the pelvis [4,7,8,9,10,11], but breathing motion in the abdomen leads to artifacts in free-breathing CBCT images that can prevent clinicians from visualizing organs and are incompatible with the auto-contouring algorithm on which Ethos relies. In light of this challenge, CT-guided adaptive radiation therapy (CTgART) workflows in the abdomen remain largely understudied. Schiff et al. [31] reported the results of a clinical imaging study in which repeated breath-hold CBCT images were acquired on Ethos for the investigation of stereotactic treatments of abdominal oligometaseses. This work was followed by the treatment of a patient with pancreatic cancer using stereotactic CTgART under breath-hold conditions [32]. Separately, Ogawa et al. [33] have explored the utility of ART on Ethos for pancreatic cancer with standard treatment volumes in a study that utilized breath-hold CBCT images acquired on a TrueBeam (Varian Medical Systems, Palo Alto, CA, USA) rather than Ethos. In the present study, we seek to evaluate the feasibility of CTgART for pancreatic cancer with a focus on two workflows: the native Ethos workflow, which relies on auto-contoured influencer structures, and a deformable image registration-based workflow that instead propagates both OAR and target contours from the planning image to each daily CBCT. This evaluation is facilitated by the creation of synthetic CBCT data derived from true, free-breathing CBCT images acquired on Ethos, which are used to conduct an in silico study of these adaptive workflows.

## 2. Materials and Methods

### 2.1. Patient Population

Ten patients were included in the present IRB-approved retrospective planning study (70% female, median age 64 years, range 53–87 years), selected from a population of patients undergoing nonadaptive treatments for pancreatic cancer on the Ethos platform in our department in the period from July to September 2022. Of the patients being treated in this period, we selected those who shared a common prescription: a total of 5040 cGy delivered to the low-dose planning target volume (PTV) with an integrated boost to 5880 cGy (only one patient’s plan did not include the high-dose target) delivered in 28 fractions. For the purposes of this study, planning CT data with clinical contours and daily setup CBCT images from the first seven treatment fractions were collected for each of the 10 patients. Clinical target volumes (CTVs) delineated by the treating physician were expanded 5 mm radial and 7 mm superior–inferior to produce the corresponding low- and high-dose PTVs; these are the same margins used for nonadaptive pancreas treatments at this institution. Critically, daily CBCT images for these nonadaptive patients treated on a standard fractionation schedule were acquired without breath-hold and no stomach filling guidelines were provided. Projections were acquired with a scanning period of 17 s and tube voltage of 125 kVp and subsequently reconstructed via an iterative CBCT reconstruction algorithm.

### 2.2. Synthetic CBCT Data

**Figure 1:**
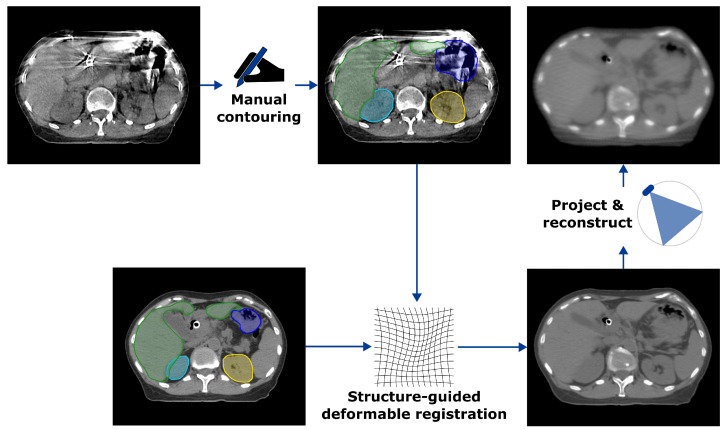
Synthetic CBCT generation process. Contours of the stomach (blue), liver (green), and both kidneys (cyan, yellow) manually drawn on daily CBCTs are used to drive a structure-guided deformable image registration from the planning CT with clinical contours to the daily CBCT. Analytical projections are then acquired of the deformed CT and reconstructed to produce the synthetic CBCT data.

The challenges stemming from motion artifacts necessitated the creation of synthetic CBCT data that did not suffer from the same streak artifacts present in the true CBCT acquisitions. Although the focus of the present study is on the evaluation of workflows for pancreatic cancer treatments in the Ethos environment rather than synthesizing image data, approaches subject to the same challenge may be of general interest [34]. Regardless, the process for creating these data is outlined in Figure 1.

Beginning with the daily CBCT images acquired on Ethos, the stomach, liver, and both kidneys were contoured in MIM 7.2.7 (MIM Software, Cleveland, OH, USA) for each of the seven treatment fractions investigated for each patient. The primary interest of the present study is related to the readily observable variations in stomach volume from day to day, but the liver and kidneys were contoured to provide additional guidance in a broader region of the body in the following step. After peer review by a physicist who covers adaptive treatments, these contours were then used to drive structure-guided deformable image registration performed in RayStation 10A (RaySearch Laboratories AB, Stockholm, SE, USA) from the planning CT (with clinical contours) to each daily CBCT for a given patient, providing an approximation of the anatomy of the day in the deformed CT images.

To better imitate the image quality of a true CBCT input to the Ethos software environment, these deformed CT images were used as the basis of analytical projection and reconstruction in RTK 2.3.0 [35] to produce synthetic CBCT data that did not contain the same detrimental streak artifacts present in the true free-breathing CBCT images acquired on Ethos. RTK utilizes Joseph forward projection [36], implemented here with the geometry of the first-generation Ethos scanner: source-to-isocenter distance 1000 mm, source-to-detector distance 1540 mm, detector offset 175 mm, and detector dimensions 1280 × 320 pixels with spacing of 0.336 × 1.344 mm. Finally, conjugate gradient reconstruction was utilized, which attempts to produce the reconstructed image *f* that minimizes

(1) $$C\left(f\right)=∥\sqrt{D}\left(Rf-p\right){∥}_{2}^{2}+{\gamma ∥\nabla f∥}_{2}^{2}+\Gamma {∥f∥}_{2}^{2},$$

with displaced detector weighting operator *D*, forward projection operator *R*, projections *p*, and weights γ=1 and Γ=500, which were chosen to minimize the norm of the difference between true and synthetic CBCT data over a test set of images from this patient population.

### 2.3. Treatment Planning

The same prescription delivered clinically in a standard fractionation scheme of 5040 cGy to the low-dose PTV with an integrated boost to 5880 cGy was employed in the present study. A common planning template was created for use with all patients; the clinical goals making up the template are listed in Table 1. Planning in the Ethos treatment planning system involves the assignment of clinical goals into categories of relative importance ranging from 1—Most Important to 4—Less Important. Individual goals are ordered within these categories to communicate the planner’s priority to the optimization engine. The optimizer in turn creates a plan quality metric composed of piecewise continuous quality functions using this set of ranked clinical goals, generating optimization structures such as non-overlapping portions of targets and critical organs and ring structures surrounding the target to further constrain the resulting dose distribution [30]. During plan optimization, the optimization engine modifies plan objectives in order to maximize the plan quality metric, beginning with changes for failures in high-priority clinical goals as specified by the planner [30]. In this way, goals such as minimum target dose are prioritized up to the specified dose level while the quality function for a mean dose goal to a critical structure, for example, drives the optimizer to continue improving this goal even beyond the specified threshold [30]. In the case of overlapping structures with conflicting goals—which is common in the setting of ART in the abdomen—the optimization engine makes use of automatically created non-overlapping optimization structures separate by gradient-controlling margins, applying the original conflicting goals to these optimization structures [30]. Again, the relative priority of the goals between and within the importance categories controls the behavior of the optimization engine in these cases; for example, the point dose constraints of GI structures are ranked above target coverage metrics in Table 1 and will thus take precedence over target coverage in the case of overlap.

Deliverable IMRT plans are created by the Automated Planning algorithm, which consists of field geometry definition, optimization, intermediate dose calculation, and leaf sequencing steps [30]. In the planning process, a set of plans is generated from pre-defined templates that include the number of fields, gantry angles, and collimator angles. In the present study, IMRT plans with 12 equidistant fields beginning at gantry angle 180° were selected for each patient.

### 2.4. Ethos Emulator and Adaptive Process

Adaptive workflows were simulated using an Ethos emulator, which allows the adaptive treatment delivery process to be performed on user-input images as a stand-in for physical CBCT acquisitions. After image acquisition, the first stage in this workflow is the creation of influencer structures—a defined set of site-specific organs that interact with common targets in a given region to influence their shape. This set includes the combined bowel, duodenum, liver, and stomach for the pancreas treatments explored here [30]. It is at this stage that the two workflows we investigate differ. The native Ethos workflow creates influencer structures via auto-contouring with a static unisex abdomen segmentation model based on a convolutional neural network, the outputs of which are post-processed to remove dislocated segments and smoothed to produce the final contours [30]. As an alternative to this, we also explore a workflow that relies entirely on deformable image registration to propagate OAR contours from the planning CT to the daily CBCT. In this case, only the bowel was left as an influencer to be created through auto-contouring while the duodenum, liver, and stomach were propagated to the daily CBCT via deformable image registration.

Following the creation of these influencer structures, targets that are not derived (i.e., the CTV) are propagated via deformable image registration from the planning CT to the daily, user-input image with the aim of producing anatomically consistent volumes [30]. Depending on the proximity to influencers, one of two approaches will be applied for the propagation of each target structure. For targets impacted by influencer motion due to their proximity, structure-guided deformable image registration is performed using a combination of normalized gradient fields as a similarity measure for edge alignment, a curvature regularizer that penalizes large deformations, and sum-of-squared differences structure guidance terms for the influencer structures that encourage the maximum overlap of the deformed and target structures [37,38]. Alternatively, for targets that are not moving with the influencers and for general organ deformation, an elastic deformation model that requires neighboring voxels to move in unison is employed [39]. In both cases, the post-processing described for auto-contour refinement is also performed on the deformed contours.

At each of the preceding steps, the user has the opportunity to edit the contours that are produced before proceeding in the workflow. Changes to the influencer structures impact the propagation of the target structures, and any edits made to the targets impact both the scheduled plan alignment and the creation of the adapted plan. In the present study, no edits were made to the influencer or target structures in either workflow to allow for a straightforward comparison of the baseline performance in the AI-based and deformation-based workflows. As such, no timing data were collected to compare this aspect of the workflows.

The final step in the adaptive process is plan selection between the scheduled plan that has been rigidly aligned to the daily image and the adapted plan that has been reoptimized in the manner previously described in Section 2.3. Dose distributions are presented for comparison alongside dose metrics and the clinical goals established during planning. The user selects the plan to be delivered, at which point clinical approval and pre-treatment quality assurance would be performed.

### 2.5. Evaluation

The primary evaluation consists of a comparison between dose metrics calculated in the scheduled and corresponding adapted plans for each of the adaptive workflows investigated here. The influencer and target contours produced in simulated fractions with each workflow were also rated in terms of clinical acceptability by a physicist who covers adaptive treatments and resident physician. A simple three-point scale was used for this purpose: 1—contours unacceptable, require major edits; 2—contours acceptable with minor edits; and 3—contours acceptable as is.

## 3. Results

### 3.1. Contour Evaluation

The interpretation of the following dosimetric results relies on the evaluation of contours produced in the auto-contouring workflow and the deformation-based workflow. In the AI-based workflow, the contours most frequently deemed clinically unacceptable by the physicist were the duodenum (11/70 fractions unacceptable), stomach (8/70), and high-dose CTV (8/63). In contrast, only minor edits to influencer structures were indicated in the vast majority of cases in the deformation-based workflow. The stomach was rated by the physicist as unacceptable in only a single fraction and the duodenum was rated unacceptable in seven fractions for a single patient by the physician.

### 3.2. Planning

**Figure 2:**
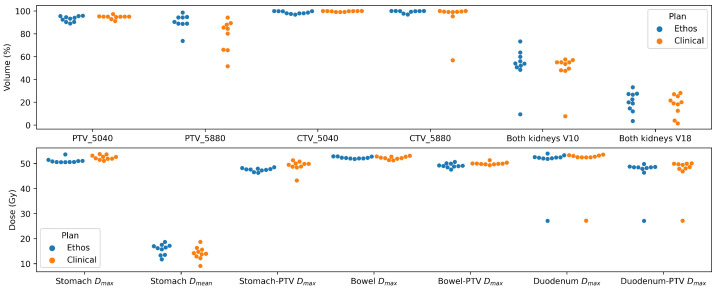
Target and OAR dose metrics achieved in the initial IMRT treatment plans (n=10) in the Ethos treatment planning system (blue) compared to the clinically delivered, nonadaptive VMAT plans (orange) for reference. Dose objectives are included in Table 1.

As a baseline point of comparison, target coverage and selected influencer dose metrics from the initial treatment plans are included in Figure 2. In these initial plans, target coverage goals were the most frequently violated among all the plan objectives, with the lowest-ranked PTV_5880 V58.80 Gy target coverage goal being satisfied in only 2 out of 10 plans. Of the constraints on OARs, kidney V10 Gy was the most frequently violated (3/10 plans) and also among the lowest-ranked objectives. Violations of dose objectives in the initial treatment plans are summarized in Table 1.

### 3.3. Dosimetric Comparisons for AI-Based Workflow

**Figure 3:**
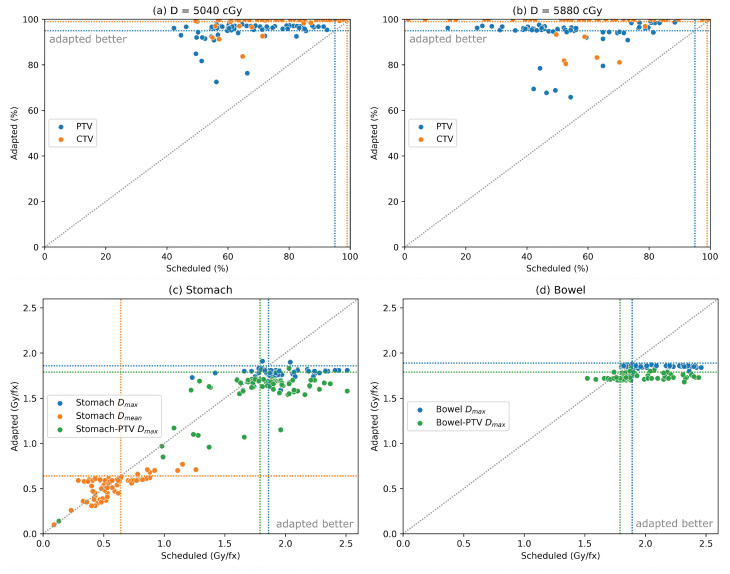

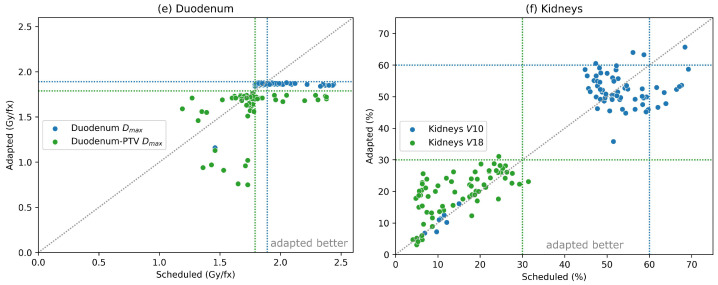
Comparison for the auto-contouring workflow between dose metrics in scheduled and adapted plans over 70 simulated fractions for the low-dose targets (**a**), high-dose targets (**b**), stomach (**c**), bowel (**d**), duodenum (**e**), and kidneys (**f**). Coverage goals and dose limits from Table 1 are illustrated in each case as dotted lines of the corresponding color along with the line of equality between scheduled and adapted plans.

Figure 3 presents dose metrics computed across 70 simulated fractions using the native AI-based Ethos adaptive workflow. In each case, a line of equality is plotted between the value achieved in the scheduled plan that was rigidly aligned to the daily image and the adapted plan that was reoptimized based on the new contours. For the low- and high-dose targets (Figure 3a,b), all points but one (CTV_5880 in Figure 3b) lie to the left of this line, indicating that plan adaptation yielded nearly uniform improvements in target coverage compared to the corresponding scheduled plans. Ultimately, low-dose PTV and CTV dose coverage goals were satisfied in 77% and 86% of adapted plans, respectively, improving from 0% and 1% of scheduled plans (unrealistic contours produced in this workflow introduce a bias in the scheduled cases). Similarly, coverage goals of the high-dose PTV and CTV were satisfied in 70% and 89% of adapted plans, compared to 0% and 16% of scheduled plans. Average improvements in target coverage between the scheduled and adapted plans are presented in Table 2. The comparison of OAR dose metrics is less uniform in terms of position relative to the line of equality, but general improvements in OAR sparing are observed with plan adaptation using the auto-contouring workflow (Figure 3c–f). Various constraints on the bowel and stomach were the most frequently violated in the scheduled plans as illustrated in Table 1 and Figure 3c,d. The average changes in OAR sparing between scheduled plans and adapted plans are summarized in Table 2. In sum, 55 adapted fractions satisfied all OAR dose constraints compared to just 5 scheduled fractions.

### 3.4. Dosimetric Comparisons for Deformation-Based Workflow

**Figure 4:**
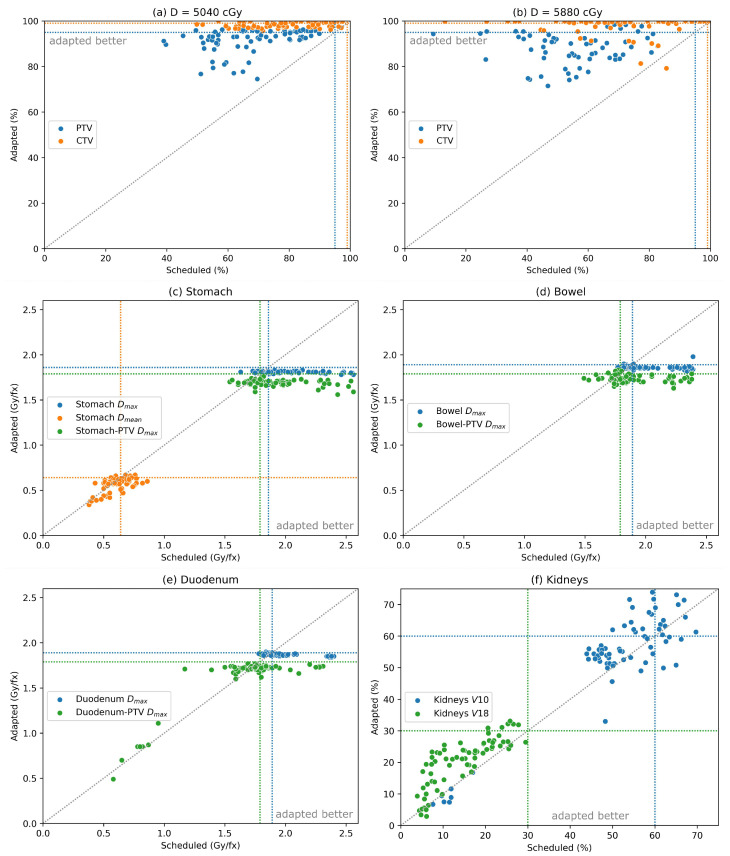
Comparison for the deformation-based workflow between dose metrics in scheduled and adapted plans over 70 simulated fractions for the low-dose targets (**a**), high-dose targets (**b**), stomach (**c**), bowel (**d**), duodenum (**e**), and kidneys (**f**). Coverage goals and dose limits from Table 1 are illustrated in each case as dotted lines of the corresponding color along with the line of equality between scheduled and adapted plans.

Figure 4 presents the same comparisons for the deformation-based workflow. There is a notable drop in target coverage in adapted fractions compared to what is observed in the AI-based workflow, although plan adaptation does offer an advantage in target coverage in the majority of cases (Figure 4a,b). Scheduled plans demonstrate similar or worse rates of failure in target coverage as in the AI-based workflow, but low-dose PTV and CTV coverage goals are satisfied in only 31% and 44% of adapted fractions, respectively. Similarly, coverage goals for the high-dose PTV and CTV are satisfied in 19% and 70% of adapted fractions. OAR constraint violations (Figure 4c–f) in this workflow occur at similar rates as in the AI-based case, with the notable exception of constraints on the kidneys as illustrated in Table 1 and Figure 4f. The drops in target coverage and increased kidney violations may be attributed to the importance assigned at the planning stage and the qualitative differences observed between the influencer contours produced in the deformation-based workflow and the auto-contouring workflow. Inferior extents of the stomach contour produced in only the deformation-based case, for example, caused the dose to be pushed into the lower-weighted kidneys in favor of sparing the stomach. Again, differences between the scheduled and adapted plans are summarized in Table 2. Like in the AI-based workflow, only 5 scheduled plans satisfied all OAR constraints in the deformation-based workflow compared to 43 adapted plans.

## 4. Discussion

**Figure 5:**
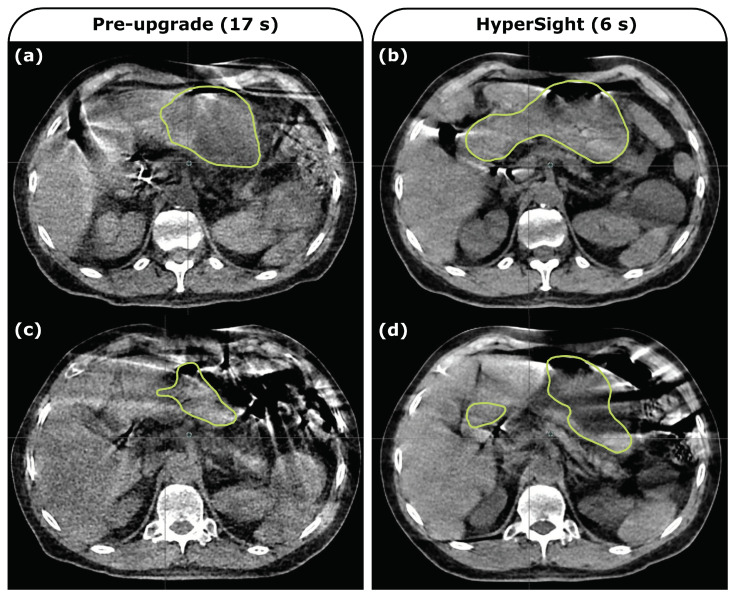
Daily CBCT images from a single patient (not included in the 10 patient cohort of this study) acquired using the pre-upgrade imaging panel with a 17 s acquisition period (left) and using the upgraded HyperSight system with a 6 s acquisition period (right). Ethos-produced stomach contours at a representative slice are shown. The anatomy observed in fractions (**a**,**b**) is similar but the full extent of the stomach is more accurately contoured in the post-upgrade image (**b**). Motion artifacts that lead to poor contouring performance as illustrated in pre-upgrade image (**c**) also cause failures post-upgrade (**d**).

CBCT-guided ART has been employed primarily in sites like the pelvis, where anatomical variation is regularly observed but motion on shorter time scales is not a primary concern. The implementation of ART in the abdomen is attractive due to the observed variability in the position of radiosensitive GI structures and has been successfully employed using MR-guided platforms, but investigations of CBCT-guided workflows have been limited. Breathing motion during the CBCT acquisition period leads to streak artifacts that are detrimental to the auto-contouring process on which the Ethos workflow relies. In light of this challenge, we have utilized synthetic CBCT data derived from true, free-breathing CBCT images acquired on Ethos to explore two adaptive workflows that differ in the approach to creating the daily contours that form the foundation of an Ethos adaptive plan. In both the native Ethos workflow that utilizes auto-contouring to produce influencer contours and the alternative workflow that instead propagates both OAR and target contours from the planning CT via deformable image registration, plan adaption demonstrates advantages in target coverage and OAR sparing, but these advantages are less pronounced in the deformation-based workflow. It is important, however, to contextualize these results in terms of the clinical acceptability of the contours produced in each case. The reliability of the auto-contoured influencers and, by extension, targets was questionable, with the duodenum and stomach contours being deemed totally unacceptable in a notable number of fractions: 16% and 11% of all fractions, respectively. Cases such as these would necessitate extensive re-contouring at a significant time cost [29]. Adopting the deformation-based workflow improved these ratings in all cases with the exception of the stomach in a single fraction, indicating that target propagation based on these more acceptable contours could be more reliable. The slight discrepancy between the physicist and physician ratings (the physician rated the duodenum unacceptable in 10% of fractions in the deformation-based workflow) illustrates a difference in priority and focus between these role groups. The physician in this case was more critical of target structures than the physicist, which may also be a product of the setting—the time pressures associated with the adaptive process in the clinic were not in place here. Along this line, a more complete understanding of how ‘ideal’ edits impact adaptive plan quality compared to ‘realistic’ edits performed under time pressure is an important topic of interest [40]. Considering again true CBCT images affected by motion artifacts that are incompatible with auto-contouring approaches, the question remains about the affect of these artifacts on the alternative approach of contour propagation via deformable registration. In both settings, it is critical to evaluate the resulting contours in place as we have carried out here with an understanding of the involved uncertainties and dosimetric impacts, for example [41]. Regardless, the general improvements observed with the deformation-based workflow highlight failure cases observed in the auto-contouring workflow. The limitations of the synthetic CBCT data utilized here feed into this issue, as the image quality resulting from the reconstruction algorithm we employ does not fully reflect what is produced in the Ethos environment, but the baseline performance of the auto-contouring algorithm as illustrated with the application to true CBCT images is also important to consider. The present study was performed using images acquired with a 17 s acquisition period as previously described, but the HyperSight imaging system upgrade consisting of hardware and software improvements has since been deployed, allowing for a 6 s acquisition period. This speed up offers improvements in image quality that can be observed to varying degrees in multiple sites, including the pancreatic cancer population of interest here. Figure 5 includes an illustrative example for a patient with images acquired both pre- and post-upgrade, demonstrating the continuing challenge of auto-contouring in this region. The model is ultimately just another observer in the contouring task rife with inter-observer variability where modifications represent a significant portion of the entire adaptive process [4,8,31,42].

Other observations from the present study highlight the challenges of pancreatic cancer treatments that make ART attractive in this setting. Achieving target coverage goals is often hampered by unacceptable overdosing of surrounding critical structures, which is a fact reflected in the priorities established during planning. We opted here to apply a template treatment plan to all patients for the sake of consistency across the cohort [9,43]. The alternative approach is to tailor the initial treatment plan for each patient, but this is an approach that implicitly relies on an assumption that planning priorities tailored to the anatomy observed at simulation are equally applicable to any variation of that anatomy observed at a later fraction. This trade-off between personalization and the flexibility or robustness of a plan highlights the challenge of planning in general in the adaptive setting, which is a topic deserving of further study. Along this line, one may reasonably expect daily differences in plan metrics to wash out over the course of treatment. The wide variation observed in mean stomach dose and dose–volume measures in the kidneys aligns with this expectation, but OAR max dose metrics in adapted plans were observed to be relatively consistent across the first seven fractions for a given patient and rarely in violation of the associated constraint. This indicates that the potential value of plan adaptation in this setting is not necessarily in achieving sparing above and beyond a constraint, but in ensuring that a constraint is met consistently throughout the course of treatment. However, this may also be a reflection of the priorities established during planning as we have already discussed. One may also consider the different impacts of these daily variations and of bulk changes that occur on a slower time scale due to tumor shrinkage or weight loss, for example. The use of data from only the first seven fractions for these patients restricts our conclusions to the impact of random daily variations, however.

Finally, it is important to note again the amount and nature of the clinical data utilized in this study. Data available from 10 previously treated patients enabled the present work but the relatively small cohort limits the generalizability of these results. Additionally, the target volumes, prescriptions, and fractionation schedules reflect the priorities of nonadaptive treatments and may not be uniformly applicable to the adaptive setting. The margin expansions applied in the creation of the PTV that yield hard-to-cover large targets, for example, may be reduced in an adaptive treatment where margin reduction is a primary aim of plan adaptation [44]. Similarly, transitioning to a hypofractionated schedule would make helpful breath-hold and stomach filling guidelines—burdensome measures that were not applied in the treatment of this patient population—more attractive or feasible for a broad patient population. Along this line, there is a need for further studies investigating realistic treatment regimes for a larger patient cohort to address questions beyond the scope of a study subject to limitations such as these.

## 5. Conclusions

Daily anatomic variation observed in the abdomen and the associated changes induced in pancreatic target structures led to poor performance by scheduled plans that introduce only rigid shifts, illustrating the potential promise of Ethos adaptive radiotherapy in this region. This promise is balanced by the requirement for artifact-reducing breath-hold images and questions regarding auto-contouring performance in the abdomen. An alternative workflow that relies on deformable image registration rather than auto-contouring to produce the daily contours may facilitate the use of the platform in this setting and help to minimize the effort devoted to refining contours in the time-sensitive adaptive process.

## Figures and Tables

**Table 1:** Clinical goals ranked by importance that make up the template plan applied to all patients. The number of violations of these goals in the initial treatment plans across all 10 patients are included along with the number of scheduled (SCH) and adapted (ADP) plans violating these goals across 70 total fractions in each of the AI-based and deformation-based workflows. One patient did not have the 5880 cGy target, so metrics for C(P)TV_5880 are out of a total of 63 fractions.

		Violations				
			AI-Based		Deformation-Based	
Structure	Objective	Planning (n=10)	SCH (n=70)	ADP (n=70)	SCH (n=70)	ADP (n=70)
1—Most Important						
Stomach	D0.25 cc ≤ 52 Gy	1	40	2	49	1
Bowel	D0.25 cc ≤ 53 Gy		37		38	3
Duodenum	D0.25 cc ≤ 53 Gy		27		28	
CTV_5040	V50.40 Gy ≥ 99%	6	69	10	70	39
CTV_5880	V58.80 Gy ≥ 99%	2	53 (of 63)	7 (of 63)	61 (of 63)	19 (of 63)
2—Very Important						
Stomach-PTV	D0.25 cc ≤ 50 Gy		43	1	48	
Bowel-PTV	D0.25 cc ≤ 50 Gy	2	54	4	46	4
Duodenum-PTV	D0.25 cc ≤ 50 Gy	2	15		26	
PTV_5040	V50.40 Gy ≥ 95%	7	70	16	70	48
PTV_5880	V58.80 Gy ≥ 95%	8	63 (of 63)	19 (of 63)	63 (of 63)	51 (of 63)
PTV_5040	V55 Gy ≤ 40%					
Stomach	$D_{mean}$ ≤ 18 Gy	1	22	7	25	4
Both kidneys	V10 Gy ≤ 60%	3	9	4	16	23
Both kidneys	V18 Gy ≤ 30%	1	1	1		6
Spinal canal	D0.25 cc ≤ 43 Gy					
Liver	V30 Gy ≤ 30%		1			
3—Important						
Liver	$D_{mean}$ ≤ 24 Gy		1			
SpinalCord_PRV5mm	D1% ≤ 43 Gy					

**Table 2:** Average differences in dose metrics between adapted plans and scheduled plans across 70 simulated fractions for the AI-based auto-contouring workflow and the deformation-based workflow.

		AI-Based	Deformation-Based
PTV_5040	V50.40 (%)	28.1±10.5	23.3±9.4
CTV_5040	V50.40 (%)	21.7±11.3	19.3±9.6
PTV_5880	V58.80 (%)	39.6±15.1	32.6±14.3
CTV_5880	V58.80 (%)	31.8±22.4	25.3±16.7
Stomach	D_max_ (cGy/fx)	−17.5±17.0	−23.2±22.6
	D_mean_ (cGy/fx)	−6.0±12.9	−4.0±7.2
Stomach-PTV	D_max_ (cGy/fx)	−23.7±18.9	−26.0±23.8
Bowel	D_max_ (cGy/fx)	−15.2±19.3	−14.5±19.1
Bowel-PTV	D_max_ (cGy/fx)	−19.8±19.0	−17.9±21.4
Duodenum	D_max_ (cGy/fx)	−15.2±20.4	−7.3±16.3
Duodenum-PTV	D_max_ (cGy/fx)	−13.8±22.4	−4.8±16.8
Both kidneys	V10 (%)	−1.4±6.6	2.8±4.8
	V18 (%)	4.6±5.1	5.6±3.7

## Data Availability

The datasets presented in this article are not readily available because they include protected health information.

## Abbreviations

The following abbreviations are used in this manuscript:

ART	Adaptive radiation therapy
OAR	Organ at risk
MRI	Magnetic resonance imaging
CT	Computed tomography
CBCT	Cone beam computed tomography
CTgART	Computed tomography-guided adaptive radiation therapy
PTV	Planning target volume
CTV	Clinical target volume
SCH	Scheduled
ADP	Adapted
